# An annotated update of the scale insect checklist of Hungary (Hemiptera, Coccoidea)

**DOI:** 10.3897/zookeys.309.5318

**Published:** 2013-06-13

**Authors:** Ferenc Kozár, Zsuzsanna Konczné Benedicty, Kinga Fetykó, Balázs Kiss, Éva Szita

**Affiliations:** 1 Plant Protection Institute, Centre for Agricultural Research, Hungarian Academy of Sciences H-1022 Budapest Herman Ottó út 15 Hungary

**Keywords:** Introduced pests, insect invasion, distribution, taxonomy, Palaearctic Region

## Abstract

The number of scale insect species (Hemiptera: Coccoidea) known from Hungary has increased in the last 10 years by 39 (16.6 %), to a total of 274 species belonging to 112 genera in10 families. The family Pseudococcidae is the most species rich, with 101 species in 34 genera; Diaspididae contains 59 species in 27 genera; Coccidae contains 54 species in 27 genera; and the Eriococcidae contains 33 species in 8 genera. The other 6 coccoid families each contain only a few species: Asterolecaniidae (7 species in 3 genera); Ortheziidae (7 species in 4 genera); Margarodidae *sensu lato* (5 species in 5 genera); Cryptococcidae (3 species in 2 genera); Kermesidae (4 species in 1genus); and Cerococcidae (1 species). Of the species in the check list, 224 were found in outdoor conditions, while 50 species occurred only in indoor conditions. This paper contains 22 species recorded for the first time in the Hungarian fauna.

## Introduction

Scale insects (Hemiptera: Coccoidea) live on a wide variety of plant species and many of them are important agricultural pests. Publication of new knowledge of this insect group is therefore very important from a practical viewpoint. The distribution data of different species may serve also as a reliable biodiversity indicator in different territories, such as nature reserves and agricultural or urban landscapes. The distribution data may also reflect the progress of climatic changes (Kozár 1997; 2009; Kozár et al. 2004; Kozár et al. 2009).

The world distribution of insect pests has changed greatly in recent decades, mainly due to increasing international trade in plant material. Scale insects are particularly well adapted to accidental introduction because their habits are often cryptic, so they can escape detection during quarantine inspections (Muniappan et al. 2009). Specifically, in recent years intensive scale insect invasions have been observed in several parts of Europe. In parallel, the number of species detected in the continent increased substantially, both outdoors and in indoor conditions such as greenhouses, commercial fruit stores and nurseries (Ben-Dov et al. 2013; Fetykó and Kozár 2012; Fetykó and Szita 2012; Malumphy and Badmin 2012; Pellizzari and Germain 2010).

Early data on the distribution of scale insects in Hungary were summarized by Kosztarab and Kozár (1978; 1988) and by Kozár (1998). The last check list of the scale insects of Hungary (Kozár 2005) reported 235 species and provided distribution maps. At the same time, the international ScaleNet database contained 206 scale insect species recorded from Hungary (Ben-Dov et al. 2013). Since the work of Kozár (2005), 17 new scale records from Hungary have been published (Fetykó and Kozár 2012; Fetykó and Szita 2012; Klupács and Volent 2012; Kozár 2004; 2009; Kozár et al. 2012; Kozár et al. in preparation; Kozár et al. 2004; Kozár and Konczné Benedicty 2005; 2007; Kozár et al. 2009). In the present paper we provide the latest checklist of scale insect species found in Hungary, and give a zoogeographic analysis of the known fauna of Central Europe and surrounding countries.

Scale insect species in Hungary are grouped into three categories. True members of the Hungarian fauna can be found regularly in outdoor habitats and typically overwinter outdoors. The second category of species is generally found in greenhouses or other buildings, mainly on ornamental plants. These introduced species are, in some cases, well-established in Hungary and may occur regularly, but are unable to overwinter outdoors. The third category consists of relatively few, introduced species that occur typically on imported tropical or subtropical fruits for consumption. Some of these species have not been able to establish at all, even in greenhouses, despite repeated introductions over several decades. All the species in the following checklist are assigned to one of these three categories.

## Materials and methods

The list below is based on the collection data of the authors between 2003 and 2013 and includes earlier records from Kozár (2005). In this ten-year period, 4738 scale insect samples were studied (Kozár’s collection index numbers 6097–0835). The samples originated from both outdoors and indoors, i.e. field trips, greenhouses, botanical gardens, nurseries, imported fruits and indoor ornamental plants.

The scales were mounted on microscope slides following the method described by Kosztarab and Kozár (1988). Voucher specimens, mainly in form of microscope slides, can be found in Kozár’s collection in the Plant Protection Institute at the Centre for Agricultural Research of the Hungarian Academy of Science.

The nomenclature of the scale insects has frequently been changed, even within the last decade. The scientific names used below therefore are annotated to relate them to those that were used in earlier Hungarian publications. We have endeavoured to maintain conformity with our previous works, as well as with the international scale insect database on “ScaleNet” (Ben-Dov et al. 2013). The taxonomic status of the families Margarodidae and Pseudococcidae are subject to current research, so these families in their wider circumscription are dicussed here as Margarodidae *sensu lato* and Pseudococcidae *sensu lato.*

For the zoogeographical and zoological subregion of Central Europe, we used the Palaearctic concept of Emeljanov (1974). Species richness data of different countries was based on the ScaleNet database (Ben-Dov et al. 2013), and published local checklists were used for comparison purposes (Fetykó et al. 2010; Foldi 2001; Gertsson 2001; Jansen 1999; Kozár 2005; Kozár et al. 1994; Kozár et al. in preparation; Lagowska 2001; Masten-Milek and Simala 2008; Pellizzari and Russo 2004; Schmutterer 2008; Seljak 2010; Tereznikova 1975; 1981; 1986).

## Results and discussion

**Figure 1.**
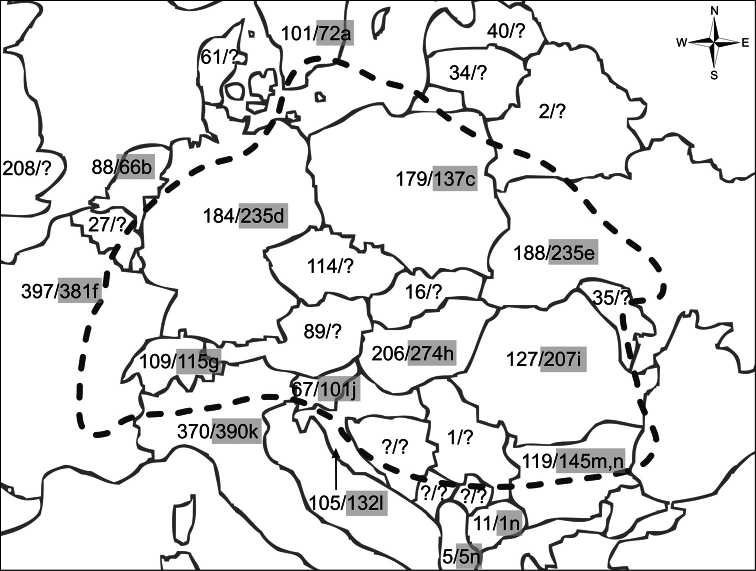
Biogeographic map of Central Europe after Emeljavon (1974) in the wider sense, with scale insect species numbers. In each country, the first number represents the number of scale insect species from ScaleNet database (Ben-Dov et al. 2013); the second number, if present, shows the number of species recorded in the following published check lists: (a) Gertsson 2001; (b) Jansen 1999; (c) Lagowska 2001; (d) Schmutterer 2008; (e) Tereznikova 1975; 1981; 1986; (f) Foldi 2001; (g) Kozár et al. 1994; (i) Fetykó et al. 2010; (j) Seljak 2010; (k) Pellizzari and Russo 2004; (l) Masten-Milek and Simala 2008; (m) Trecheva et al. 2012; (n) Tomov et al. 2009 and the present work for Hungary (h).

The number of scale insect species in Hungary has increased by 39 (16.6 %) in the last ten years, and currently totals 274 species in ten families (Tables 1 and 2). The largest families in order of species richness are: Pseudococcidae with 101 species, Diaspididae (59 species), Coccidae (54 species) and Eriococcidae with 33 species. The new species to the Hungarian fauna recorded here belong to the Pseudococcidae, Diaspididae and Eriococcidae. Most of the species in the checklist (224; 81.75 %) are native and live outdoors. The check list contains 50 introduced (generally cosmopolitan) species, mainly occurring indoors in Hungary on ornamental plants in greenhouses and buildings. Of these indoor species, 33 occurred only in greenhouses or buildings (mainly on ornamental plants) and 7 were found exclusively on imported tropical/subtropical fruits for consumption. Four of the species living in greenhouses sometimes also occur outdoors. Four other species, which are typically found on imported fruit, also appear in greenhouses from time to time. Two of the newly recorded species were found on imported nursery plant material. In the present list, 22 species are new to the Hungarian fauna. According to these data, Hungary is the most scale-insect-species-rich country in in Central Europe (Fig. 1).

**Table 1.** The number of scale insect species in different categories.<br/>

	**Number of species**	**%**
New to the Hungarian fauna	22	8.03
Only found outdoors	224	81.75
Introduced on propagation plant material (outdoor conditions)	2	0.73
Only found indoors (in greenhouses and buildings)	33	12.40
Only found on imported fruit	7	2.55
Mainly found in greenhouses	4	1.46
Mainly on imported fruits, but occasionally in greenhouses	4	1.46
Total	274	-

**Table 2.** Updated checklist of scale insects (Homoptera: Coccoidea) of Hungary (2013), with comments and nomenclatural changes. Information on the original decriptions of species can be found in ScaleNet database (Ben-Dov et al. 2013).<br/>

**Taxon**	**Comment**
**Asterolecaniidae** (3 genera)	
*Asterodiaspis bella* (Russell, 1941)	
*Asterodiaspis quercicola* (Bouché, 1851)	
*Asterodiaspis roboris* (Russell, 1941)	
*Asterodiaspis variolosa* (Ratzeburg, 1870)	
*Asterodiaspis viennae* (Russell, 1941)	
*Asterolecanium epidendri* (Bouché, 1844)	
*Planchonia arabidis* Signoret, 1877	Previously recorded as *Asterolecanium fimbriatum* (Leonardi, 1920).
**Cerococcidae** (1 genus)	
*Cerococcus cycliger* Goux, 1932	
**Coccidae** (27 genera)	
*Ceroplastes japonicus* Green, 1921	Found in Hungary in 2011 (Klupács and Volent 2012).
*Ceroplastes rubens* Maskell, 1893	Found in Hungary in 2011 (Fetykó and Kozár 2012).
*Ceroplastes rusci* (Linnaeus, 1758).	According to Kosztarab (1955) the latest record of this species in Hungary was published in 1883. Has probably disappeared.
*Chloropulvinaria floccifera* (Westwood, 1870)	
*Coccus hesperidum* Linnaeus, 1756	Found on *Maclura* sp. outdoors in recent years in Hungary (Velence) (Salamon and Tőkés 2010). Overwintering method not known.
*Eriopeltis festucae* (Fonscolombe, 1834)	
*Eriopeltis lichtensteini* Signoret, 1876	
*Eriopeltis stammeri* Schmutterer, 1952	
*Etiennea villiersi* Matile-Ferrero, 1984	It has not been found since its first record (Kozár 2005), and has probably disappeared.
*Eucalymnatus tessellatus* (Signoret, 1873)	
*Eulecanium ciliatum* (Douglas, 1891)	
*Eulecanium franconicum* (Lindinger, 1912)	
*Eulecanium tiliae* (Linnaeus, 1758)	Previously recorded as *Eulecanium mali* (Schrank, 1781).
*Eupulvinaria hydrangeae* (Steinweden, 1946)	
*Exaeretopus formiceticola* Newstead, 1894	
*Exaeretopus mahunkai* Kozár & Drozdják, 1990	
*Gascardia hodgsoni* Matile-Ferrero & Le Ruyet, 1985	It has not been found since its first record (Kozár 2005), and has probably disappeared.
*Lecanopsis formicarum* Newstead, 1893	Previously recorded as *Lecanopsis terrestris* Borchsenius, 1952.
*Lecanopsis subterranea* (Gomez Menor Ortega, 1948	Previously recorded as *Longicoccus festucae* Borchsenius, 1952.
*Lecanopsis turcica* (Bodenheimer, 1951)	Previously recorded as *Lecanopsis porifera* Borchsenius, 1952
*Luzulaspis kosztarabi* Koteja & Kozár, 1979	
*Luzulaspis nemorosa* Koteja, 1966	Previously recorded as *Lecanopsis luzulae* (Dufour, 1864).
*Luzulaspis rajae* Kozár, 1981	
*Luzulaspis scotica* Green, 1926	Previously recorded as *Lecanopsis borchsenii* Rehacek 1959.
*Palaeolecanium bituberculatum* (Targioni Tozzetti, 1868)	
*Parafairmairia bipartita* (Signoret, 1872)	
*Parafairmairia gracilis* Green, 1916	
*Parthenolecanium corni* (Bouché, 1844)	
*Parthenolecanium fletcheri* (Cockerell, 1893)	
*Parthenolecanium persicae* (Fabricius, 1776)	
*Parthenolecanium pomeranicum* (Kawecki, 1954)	
*Parthenolecanium rufulum* (Cockerell, 1903)	
*Phyllostroma myrtilli* (Kaltenbach, 1874).	The occurrence of this species in Hungary was mentioned by Lindinger (1912). No voucher specimen in Kozár’s collection.
*Physokermes hemicryphus* (Dalman, 1826)	
*Physokermes inopinatus* Danzig & Kozár, 1973	
*Physokermes piceae* (Schrank, 1801)	
*Poaspis intermedia* (Goux, 1939)	Previously recorded as *Luzulaspis*.
*Poaspis jahandiezi* (Balachowsky, 1932)	Previously recorded as *Luzulaspis*.
*Poaspis lata* (Goux, 1939)	
*Psilococcus ruber* Borchsenius, 1952	
*Pulvinaria ribesiae* Signoret, 1873	
*Pulvinaria vitis* (Linnaeus, 1758)	Previously recorded as *Pulvinaria betulae* (Linnaeus, 1758).
*Pulvinariella mesembryanthemi* (Vallot, 1830)	It has not been found since its first record (Kozár 2005), and has probably disappeared.
*Rhizopulvinaria artemisiae* (Signoret, 1873)	
*Rhizopulvinaria gracilis* Canard, 1967	
*Rhizopulvinaria spinifera* Borchsenius, 1952	
*Rhodococcus perornatus* (Cockerell & Parrott, 1899)	Previously recorded as *Rhodococcus bulgariensis* (Wünn, 1939) or *Rhodococcus rosophilus* Borchsenius, 1953.
*Rhodococcus spireae* (Borchsenius, 1949)	
*Saissetia coffeae* (Walker, 1852)	Previously recorded as *Saissetia hemisphaerica* (Targioni Tozzetti, 1867).
*Saissetia oleae* (Olivier, 1791)	
*Scythia craniumequinum* Kiritchenko, 1938	
*Scythia festuceti* (Šulc, 1941)	
*Sphaerolecanium prunastri* (Fonscolombe, 1834)	
*Vittacoccus longicornis* (Green, 1916)	
**Cryptococcidae** (2 genera)	
*Cryptococcus aceris* Borchsenius, 1937	
*Cryptococcus fagisuga*, Lindinger, 1912	
*Pseudochermes fraxini* (Kaltenbach, 1860)	
**Diaspididae** (27 genera)	
*Abgrallaspis cyanophylli* (Signoret, 1869)	Found in 2013 by K. Fetykó on *Globularia punctata* outdoors in Hungary (Budapest, Sashegy). Overwintering method unknown.
*Aonidia lauri* (Bouché, 1833)	
*Acanthomytilus jablonowskii* Kozár & Matile-Ferrero, 1983	
*Aonidiella aurantii* (Maskell, 1879)	
*Aspidiotus nerii* Bouché, 1833	Previously recorded as *Aspidiotus hederae* Signoret, 1869.
*Aspidiotus destructor* Signoret, 1869	New to the Hungarian fauna. Found in 2013 by K. Fetykó on *Phoenix roubellini* indoors in Hungary (Kecskemét).
*Aulacaspis rosae* (Bouché, 1833)	
*Aulacaspis yatsumatsui* Takagi, 1977	New to the Hungarian fauna. Found in 2012 by K. Fetykó on *Cycas revoluta* indoors in Hungary (Kecskemét).
*Carulaspis carueli* (Signoret, 1869)	New to the Hungarian fauna. Found in 2009-2012 by F. Kozár indoors and outdoors in Hungary (Csepel, Nagykovácsi, Solymár, Zalakomár), on nursery plants (*Thuja* sp., *Chamaecyparis* sp., *Juniperus* sp.).
*Carulaspis juniperi* (Bouché, 1851)	In high densities on ornamental plants in recent years.
*Carulaspis visci* (Schrank, 1781)	According to Kosztarab (1955) the first record of the species in Hungary was in 1950. No voucher specimen in Kozár’s collection.
*Chionaspis austriaca* Lindinger, 1912	According to Kosztarab (1955) the first record of the species in Hungary was in 1938. No voucher specimen in Kozár’s collection.
*Chionaspis lepineyi* Balachowsky, 1928	
*Chionaspis salicis* (Linnaeus, 1958)	
*Chortinaspis subterraneus* (Lindinger, 1912)	
*Chrysomphalus aonidum* (Linnaeus, 1758)	Previously recorded as *Chrysomphalus ficus* Ashmead, 1880.
*Chrysomphalus dictyospermi* (Morgan, 1889)	
*Diaspidiotus alni* (Marchal, 1909)	
*Diaspidiotus bavaricus* (Lindinger, 1912)	
*Diaspidiotus gigas* (Thiem & Gerneck, 1934)	Previously recorded as *Quadraspidiotus*.
*Diaspidiotus labiatarum* (Marchal, 1909)	Previously recorded as *Quadraspidiotus*.
*Diaspidiotus lenticularis* (Lindinger, 1912)	Previously recorded as *Quadraspidiotus*.
*Diaspidiotus marani* Zahradnik, 1952	Previously recorded as *Quadraspidiotus*.
*Diaspidiotus ostreaeformis* (Curtis, 1843)	Previously recorded as *Quadraspidiotus*.
*Diaspidiotus perniciosus* (Comstock, 1881)	Previously recorded as *Quadraspidiotus*.
*Diaspidiotus pyri* (Lichtenstein, 1881)	Previously recorded as *Quadraspidiotus*.
*Diaspidiotus sulci* Balachowsky, 1950	Previously recorded as *Quadraspidiotus*.
*Diaspidiotus wuenni* (Lindinger, 1923)	
*Diaspidiotus zonatus* (Frauenfeld, 1868)	Previously recorded as *Quadraspidiotus*; *Diaspidiotus hungaricus* Kosztarab, 1956 is a synonym.
*Diaspis bouisduvali* Signoret, 1869	New to the Hungarian fauna. Found in 2006 by É. Szita on *Ananas* sp. indoors in Hungary (Budapest).
*Diaspis bromeliae* (Kerner, 1778)	
*Diaspis echinocacti* (Bouché, 1833)	
*Dynaspidiotus abietis* (Schrank, 1776)	Previously recorded as *Nuculaspis*.
*Dynaspidiotus britannicus* (Newstead, 1896)	
*Epidiaspis leperii* (Signoret, 1869)	
*Ferreroaspis hungaricus* (Vinis, 1981)	Previously recorded as *Acanthomytilus*
*Hemiberlesia rapax* (Comstock, 1881)	
*Lepidosaphes beckii* (Newman, 1869).	Often in high densities on imported lemon and orange fruit.
*Lepidosaphes conchiformis* (Gmelin, 1789)	Previously recorded as *Mytilaspis rubri* Thiem, 1931.
*Lepidosaphes gloverii* (Packard, 1869)	
*Lepidosaphes granati* Koroneos, 1934	Previously recorded as *Mytilococcus*.
*Lepidosaphes newsteadi* (Šulc, 1895)	
*Lepidosaphes ulmi* (Linnaeus, 1758)	The validity of the synonyms *Lepidosaphes tiliae* Savescu, 1957 and *Lepidosaphes populi* Savescu, 1957 and/or their presence in Hungary is questionable.
*Leucaspis loewi* Colvée, 1882	Previously recorded as *Anamaspis*. In very high densities in recent years (Kozár et al. 2012).
*Leucaspis pini* (Hartig, 1839).	In very high densities in recent years (Kozár et al. 2012).
*Leucaspis pusilla* Löw, 1883.	In very high densities in recent years (Kozár et al. 2012).
*Mohelnaspis massiliensis* (Goux, 1937)	
*Mycetaspis personata* (Comstock, 1883)	
*Parlatoria crotonis* Douglas, 1887	
*Parlatoria pergandii* Comstock, 1881	
*Parlatoria ziziphi* (Lucas, 1853)	
*Pinnaspis aspidistrae* (Signoret, 1869)	
*Pinnaspis strachani* (Cooley, 1899)	
*Pseudaulacaspis pentagona* (Targioni Tozzetti, 1886)	Important outdoor pest of fruit and and ornamental plantss in Hungary; found in 2012 by K. Fetykó on kiwi fruit imported from Greece.
*Rhizaspidiotus balachowskyi* Kozár & Matile-Ferrero, 1983	
*Syngenaspis parlatoriae* Šulc, 1895	Placed by some authors in *Parlatoria*.
*Targionia vitis* (Signoret, 1876)	
*Unaspis euonymi* (Comstock, 1881)	A very important pest of *Euonymus* in towns in recent years.
*Unaspis yanonensis* (Kuwana, 1923)	New to the Hungarian fauna. Found in 2013 in Hungary (Budapest) indoors.
	
**Eriococcidae** (8 genera)	
*Acanthococcus aceris* Signoret, 1875	
*Acanthococcus melnikensis* Hodgson & Trencheva, 2008	New to the Hungarian fauna. Found in 1969 in Hungary (Vászoly) by F. Kozár on *Quercus* sp.
*Acanthococcus roboris* (Goux, 1931)	
*Acanthococcus thymi* (Schrank, 1801)	
*Anophococcus agropyri* Borchsenius, 1949	Previously recorded as *Acanthococcus* or *Rhizococcus*.
*Anophococcus cingulatus* (Kiritchenko, 1940)	Previously recorded as *Acanthococcus* or *Rhizococcus*.
*Anophococcus cynodontis* (Kiritchenko, 1940)	Previously recorded as *Acanthococcus* or *Rhizococcus*.
*Anophococcus granulatus* (Green, 1931)	New to the Hungarian fauna. Found in 2007 in Hungary (Vászoly) by F. Kozár on Poaceae.
*Anophococcus herbaceus* Danzig, 1962	Previously recorded as *Acanthococcus* or *Rhizococcus*.
*Anophococcus insignis* (Newstead, 1891)	Previously recorded as *Acanthococcus* or *Rhizococcus*.
*Anophococcus* species nova Kozár & Konczné Benedicty, 2013	Previously recorded as *Rhizococcus cistacearum* (Goux, 1936), a misidentification of *Anophococcus* sp. n.). New to the Hungarian fauna. Found in 2008 in Hungary (Fehérszék) by G. Konz on *Festuca* sp.
*Anophococcus pseudinsignis* (Green, 1921)	Previously recorded as *Acanthococcus* or *Rhizococcus*.
*Gossyparia spuria* (Modeer, 1778)	
*Greenisca brachypodii* Borchsenius & Danzig, 1966	
*Greenisca gouxi* (Balachowsky, 1954)	
*Gregoporia erwini* Kozár, 1996	Previously recorded as *Greenisca*.
*Kaweckia glyceriae* (Green, 1921)	Previously recorded as *Greenisca*.
*Kaweckia laeticoris* (Tereznikova, 1965)	Previously recorded as *Greenisca*.
*Ovaticoccus agavium* (Douglas, 1888)	
*Rhizococcus artiguesi* Goux, 1991	New to the Hungarian fauna. Found in 2011 in Hungary (Budaörs) by F. Kozár on *Thymus glabrescens*.
*Rhizococcus baldonensis* Rasina, 1966	
*Rhizococcus cantium* (Williams, 1985)	Previously recorded as *Acanthococcus*.
*Rhizococcus echinatus* (Goux, 1936)	New to the Hungarian fauna. Found by D-vac method in 1982 in Hungary (Sashegy) by A. Rákóczi, on *Festucetum*.
*Rhizococcus desertus* Matesova, 1957	Previously recorded as *Acanthococcus*.
*Rhizococcus devoniensis* (Green, 1896)	Previously recorded as *Acanthococcus*.
*Rhizococcus gnidii* Silvestri, 1875	New to the Hungarian fauna. Found in 1981 in Hungary (Budaörs) by F. Kozár on *Thymus glabrescens*.
*Rhizococcus greeni* (Newstead, 1898)	
*Rhizococcus istresianus* (Goux, 1989)	New to the Hungarian fauna. Found in 2007 in Hungary (Törek) by F. Kozár on *Hieracium* sp.
*Rhizococcus micracanthus* Danzig, 1975	Previously recorded as *Acanthococcus*.
*Rhizococcus munroi* Boratynski, 1962	Previously recorded as *Acanthococcus*.
*Rhizococcus reynei* (Schmutterer, 1952)	Previously recorded as *Acanthococcus*.
*Rhizococcus targassoniensis* (Goux, 1993)	New to the Hungarian fauna. Found in 2008 in Hungary (Bócsa) by Z. Konczné Benedicty on *Artemisia* sp.
*Rhizococcus zernae* (Tereznikova, 1977)	New to the Hungarian fauna.
**Kermesidae** (1 genus)	
*Kermes bacciformis* Leonardi, 1908	
*Kermes gibbosus* Signoret, 1875	
*Kermes quercus* (Linnaeus, 1758)	
*Kermes roboris* (Fourcroy, 1785)	
**Margarodidae** (5 genera)	
*Dimargarodes mediterraneus* Silvestri, 1906	
*Icerya purchasi* (Maskell, 1878)	
*Matsucoccus pini* (Green, 1925)	Previously recorded as *Matsucoccus matsumurae* (Kuwana, 1905).
*Neomargarodes festucae* Archangelskaja, 1935	
*Porphyrophora polonica* (Linnaeus, 1758)	
**Ortheziidae** (4 genera)	
*Insignorthezia insignis* (Browne, 1887)	Previously recorded as *Orthezia*.
*Newsteadia floccosa* (De Geer, 1778)	
*Orthezia arenariae* Vayssiere, 1923	
*Orthezia urticae* (Linnaeus, 1758)	
*Orthezia yashusi* Kuwana, 0923	
*Ortheziola britannica* Kozár & Miller, 2000	
*Ortheziola vejdovskyi* Šulc, 1895	
**Pseudococcidae** (35 genera)	
*Atrococcus achilleae* (Kiritchenko, 1936)	
*Atrococcus arakelianae* (Ter-Grigorjan, 1964)	
*Atrococcus bejbienkoi* Kozár & Danzig, 1976	
*Atrococcus cracens* Williams, 1962	
*Atrococcus paludinus* (Green, 1921)	
*Balanococcus boratynskii* Williams, 1962	
*Balanococcus singularis* Schmutterer, 1952	Previously recorded as *Trionymus*.
*Boreococcus ingricus* Danzig, 1960	
*Brevennia pulveraria* (Newstead, 1892)	
*Ceroputo pilosellae* (Šulc, 1898)	Previously recorded as *Puto*.
*Chaetococcus phragmitis* (Marchal, 1909)	
*Chaetococcus sulci* (Green, 1934)	
*Chnaurococcus danzigae* Kozár & Kosztarab, 1976	
*Chorizococcus rostrellum* (Lobdell, 1930)	
*Chorizococcus senarius* McKenzie, 1967	Previously this species was known only from USA (California) (Ben-Dov et al. 2013). It was found in Hungary (Töreki) at a highway rest area on *Cynodon dactylon*. The mealybug could have be introduced on transported plant material.
*Coccidohystrix samui* Kozár & Benedicty, 1997)	
*Coccura comari* (Künow, 1880)	
*Dysmicoccus brevipes* (Newstead, 1891)	
*Dysmicoccus walkeri* (Newstead, 1891)	
*Fonscolombia europeae* (Newstead, 1897)	
*Fonscolombia graminis* Lichtenstein, 1877	
*Fonscolombia tomlini* (Newstead, 1892)	Previously recorded as *Phenacoccopsis*.
*Geococcus coffeae* Green, 1933	
*Heliococcus bohemicus* Šulc, 1912	
*Heliococcus danzigae* Bazarov, 1974	
*Heliococcus glacialis* (Newstead, 1900)	Previously recorded as *Heliococcus cydoniae* Borchsenius, 1937.
*Heliococcus radicicola* Goux, 1934	
*Heliococcus salviae* Borchsenius, 1949	
*Heliococcus sulci* Goux, 1934	
*Heterococcus agropyri* Savescu, 1985	Proposed as a synonym of *Heterococcus nudus* (Green, 1926).
*Heterococcus nudus* (Green, 1926)	
*Heterococcus tritici* (Kiritchenko, 1932)	
*Kissrhizoecus hungaricus* Kozár & Konczné Benedicty, 2004	An element from the steppes, origin unknown.
*Longicoccus ashtarakensis* Ter-Grigorjan, 1964	New to the Hungarian fauna. Found in 2004 in Hungary (Orgovány) by F. Kozár and Z. Konczné Benedicty, on *Festuca* sp. Probably native.
*Longicoccus festucae* (Koteja, 1971)	
*Longicoccus psammophilus* (Koteja, 1971)	
*Metadenopus festucae* Šulc, 1933	
*Mirococcopsis avetianae* Ter-Grigorian, 1964	
*Mirococcopsis borchsenii* (Ter-Grigorian, 1964)	Previously recorded as *Eumirococcus*.
*Mirococcopsis elongatus* Borchsenius, 1948	
*Mirococcopsis nagyi* Kozár, 1981	
*Mirococcopsis subterraneus* (Newstead, 1893)	Previously recorded as *Chnaurococcus*.
*Nipaecoccus nipae* (Maskell, 1892)	
*Peliococcus balteatus* (Green, 1928)	
*Peliococcus chersonensis* (Kiritchenko, 1935)	
*Peliococcus marrubii* (Kiritchenko, 1935)	Previously recorded as *Spinococcus*.
*Peliococcus rosae* Danzig, 2001	Previously recorded as *Spinococcus morrisoni* Kiritchenko, 1935.
*Peliococcus turanicus* (Kiritchenko, 1931)	
*Pelizzaricoccus gabrielis* Kozár, 1991	New to theHungarian fauna. Found in 2005 by D-vac in Hungary (Nagykovácsi), by F. Samu and E. Botos. Origin unknown.
*Phenacoccus abditus* Borchsenius, 1949	
*Phenacoccus aceris* (Signoret, 1875)	Previously recorded by the synonym *Phenacoccus mespili* (Signoret, 1875). No voucher specimens available.
*Phenacoccus avenae* Borchsenius, 1949	
*Phenacoccus bicerarius* Borchsenius, 1949	
*Phenacoccus evelinae* (Tereznikova, 1975)	Previously recorded as *Paroudablis graminis* Tereznikova, 1968.
*Phenacoccus ferulae* Borchsenius, 1949	
*Phenacoccus hordei* (Lindeman, 1886)	
*Phenacoccus interruptus* Green, 1923	Previously recorded as *Paroudablis*.
*Phenacoccus persimplex* Borchsenius, 1949	
*Phenacoccus phenacoccoides* (Kiritchenko, 1932)	
*Phenacoccus piceae* Löw, 1883	Previously recorded as *Paroudablis*.
*Phenacoccus pumilus* Kiritchenko, 1935	
*Planococcus citri* (Risso, 1813)	In high densities in greenhouses and buildings; males were caught by pheromone traps in Central Europe.
*Planococcus vovae* (Nassonov, 1908)	Previously recorded as *Allococcus*. In high densities in recent years on *Thuja* sp., *Juniperus* sp., and *Chamaecyparis* sp. (Fetykó 2010).
*Polystomophora ostiaplurima* (Kiritchenko, 1940)	
*Pseudococcus elisae* Borchsenius, 1947	New to the Hungarian fauna. Found in 2007 in Hungary (Gyál) by K. Fürst on *Musa* sp. fruits. Unknown origin.
*Pseudococcus longispinus* (Targioni Tozzetti, 1868)	Previously recorded as *Pseudococcus microadonidum*.
*Pseudococcus microadonidum* Beardsley, 1966	
*Pseudococcus viburni* (Signoret, 1875)	Previously recorded as *Pseudococcus affinis* (Maskell, 1894), *Pseudococcus obscurus* Essig, 1909, or *Pseudococcus maritimus* Ehrhorn, 1900).
*Puto superbus* (Leonardi, 1907)	Previously recorded as *Macrocerococcus*.
*Rhizoecus albidus* Goux, 1936)	
*Rhizoecus cacticans* (Hambleton, 1946)	
*Rhizoecus falcifer* Künckel d’Herculais, 1878	
*Rhizoecus franconiae* Schmutterer, 1956	
*Rhizoecus kazahstanus* Matesova, 1980	
*Rhodania porifera* Goux, 1935	
*Ripersiella caesii* Schmutterer, 1956	New to the Hungarian fauna. Found in 2007 in Hungary (Sárbogárd) by B. Kiss on *Festuca* sp. Probably native. Previously recorded as *Rhizoecus*.
*Ripersiella halophila* (Hardy, 1868)	Previously recorded as *Rhizoecus*.
*Ripersiella lelloi* (Mazzeo, 1995)	
*Ripersiella periolana* (Goux, 1985)	Previously recorded as *Ripersiella halophilus*.
*Ripersiella poltavae* Laing, 1929	Previously recorded as *Rhizoecus*.
*Ritsemia pupifera* Lichtenstein, 1879	
*Spilococcus artemisiphilus* Tang, 1988	New to the Hungarian fauna. Found in 2009 in Hungary (Csepel) by F. Kozár and É. Szita on *Lotus corniculatus*. Probably native.
*Spilococcus furcatissispinus* (Borchsenius, 1937)	New to the Hungarian fauna. Found in 2009 in Hungary (Lajosmizse) by F. Kozár on *Festuca* sp. Probably native.
*Spilococcus halli* (McKenzie & Williams, 1965)	Previously recorded as *Chorizococcus viktorina*.
*Spilococcus mamillariae* (Bouché, 1844)	Previously recorded as *Spilococcus cactearum*.
*Trionymus aberrans* Goux, 1938	
*Trionymus dactylis* Green, 1925	
*Trionymus elymi* (Borchsenius, 1949)	
*Trionymus graminellus* Borchsenius, 1949	New to the Hungarian fauna. Found in 2010 in Hungary (Törökbálint) by F. Kozár on *Festuca* sp. Probable native.
*Trionymus hamberdi* (Borchsenius, 1949)	
*Trionymus multivorus* (Kiritchenko, 1935)	
*Trionymus newsteadi* (Green, 1917)	
*Trionymus perrisii* (Signoret, 1875)	
*Trionymus phalaridis* Green, 1925	
*Trionymus radicum* (Newstead, 1895)	
*Trionymus singularis* Schmutterer, 1952	New to the Hungarian fauna. Found in Hungary (Gyál) by F. Kozár on *Agropyron sp*. Probably native.
	
*Trionymus thulensis* Green, 1931	
*Trionymus tomlini* Green, 1925	
*Volvicoccus stipae* Borchsenius, 1949	Previously recorded as *Mirococcopsis*.
*Volvicoccus volvifer* (Goux, 1945)	New to the Hungarian fauna. Found in Hungary (Sashegy) by D-vac (leg: E. Botos) on *Brometum*. Probably native.
*Vryburgia brevicruris* (McKenzie, 1960)	

Comments:

i. The record of the presence of *Acanthomytilus sacchari* (Hall, 1923) in Hungary was given by Danzig and Pellizzari in Kozár (ed.) (1998), cited by ScaleNet, is not proven.

ii. The presence of *Lepidosaphes shanxiensis* Shi, 1990 in Hungary, cited by ScaleNet, is not proven (error or misidentification).

iii. The record of the presence of *Parlatoria oleae* (Colvée, 1880) in Hungary given by Kosztarab and Kozár (1988), based on US quarantine record cited by ScaleNet, is not proven.

iv. The record of *Kermes ilicis* (Linnaeus, 1758) given by Sugonyaev (1965) as a host of a parasitoid, cited by ScaleNet as a scale distribution record, is a misunderstanding of the text; the distribution record concerns the parasitoid species, not the scale.

v. The record of the presence of *Luzulaspis frontalis* Green, 1928, cited by ScaleNet as a scale distribution record for Hungary, is probably a misunderstanding of the text of Kosztarab and Kozár (1978), where it was mentioned as possibly present in Hungary.

No species should be considered as truly endemic only on the basis of its presence in a checklist, because the lack of a species in the surrounding countries is most likely due to inadequate exploration of those areas (Fig. 1). Out of the above list, 106 (38.69%) species are considered as widely distributed Pan-Palaearctic species, 75 (27.37%) are widely distributed Euro-Siberian species, 91 (33.21%) are cosmopolitan, and only two species are known to originate from the Mediterranean subregion.

Our data from Hungary shows a substantially different picture from that of earlier analyses dealing with scale insect zoogeography (Danzig 1980; Kozár 1995; Kozár and Drozdják 1986), where most of the species were thought to be restricted to one of the special subregions of the Palaearctic Region. The high proportion of the Palaearctic and cosmopolitan species in our analysis shows some similarity with the fauna of Israel (Ben-Dov 2012). This may be explained by the special borderline situation of each of these countries. Both are situated on the borders between different zoogeographic regions: Hungary on the borders of the European-Siberian and Mediterranean subregions, with strong influence from Irano-Turanian subregions; while Israel is on the borders of the Palaearctic, Oriental and the Ethiopian regions. Hungary has a temperate climate, but with several submediterranean, xerophilous habitats. In addition, the Great Hungarian Plain belongs to the steppic province of the Palaearctic Region, which ranges from Hungary to China and the Far East (Emeljanov 1974). The importance of the steppic influence can be seen in that almost 50% of the species belong to the families Pseudococcidae and Eriococcidae, and most of them live on grasses and small herbaceous plants (Table 3).

**Table 3.** Number of scale insect species in Hungary, by family<br/>

**Family**	**Number of species**	**% of the Hungarian fauna**	**Number of new records**
Asterolecaniidae	7	2.55	0
Cerococcidae	1	0.36	0
Coccidae	54	19.71	0
Cryptococcidae	3	1.09	0
Diaspididae	59	21.53	5
Eriococcidae	33	12.04	9
Kermesidae	4	1.46	0
Margarodidae s.l.	5	11.82	0
Ortheziidae	7	2.55	0
Pseudococcidae	101	36.86	8

In Central Europe in the wide bio-geographic sense of Emeljanov (1974), the Hungarian list of 274 species (Fig. 1) represents the greatest species-richness value among the component countries. Undoubtedly this is partly due to better exploration of Hungary. However, it is also due to the various climatic influences affecting the territory from different directions. Important differences in species richness values were found between the data on ScaleNet and the local checklists (Fetykó et al. 2010; Foldi 2001; Gertsson 2001; Jansen 1999; Kozár 2005; Kozár et al. 1994; Kozár et al. in preparation; Lagowska 2001; Masten-Milek and Simala 2008; Pellizzari and Russo 2004; Schmutterer 2008; Seljak 2010; Tereznikova 1975; 1981; 1986, Tomov et al. 2009; Trencheva 2012). The map shows that some countries, like Bosnia and Serbia, are inadequately represented in the ScaleNet database. The published check lists in general show a more reliable picture; however, in some cases we meet an opposite situation (for example, for Germany and France). These discrepancies need further study in the future.

Concerning the category of species found indoors in greenhouses and buildings (Table 4) detailed information on these species are available in Kosztarab and Kozár (1978; 1988), in Kozár (1989; 1998; 2005) and on ScaleNet. Four species in this category are new records for the Hungarian fauna. (*Diaspis bouisduvali* Signoret, 1869, *Unaspis yanonensis* (Kuwana, 1923), *Aspidiotus destructor* Signoret, 1869, *Aulacaspis yatsumatsui* Takagi, 1977). The following species have become significant pests in Hungary in recent years: *Aspidiotus nerii* Bouché, 1833; *Coccus hesperidum* Linnaeus, 1758; *Planococcus citri* (Risso, 1813); *Pseudococcus longispinus* (Targioni Tozzetti, 1868); *Pseudococcus viburni* (Signoret, 1875) and *Saissetia coffeae* (Walker, 1852) (Kozár 1989).

**Table 4.** List of scale insect species found indoors in greenhouses and buildings (on ornamental plants) in Hungary<br/>

*Abgrallaspis cyanophylli* (Signoret, 1869)
*Aonidia lauri* (Bouché, 1833)
*Aspidiotus nerii* Bouché, 1833
*Aspidiotus destructor* Signoret, 1869.
*Asteroleanium epidendri* (Bouché, 1844)
*Aulacaspis yatsumatsui* Takagi, 1977
*Ceroplastes japonicus* Green, 1921
*Ceroplastes rubens* Maskell, 1893
*Ceroplastes rusci* (Linnaeus, 1758)
*Chrysomphalus aonidum* (Linnaeus, 1758)
*Chrysomphalus dictyospermi* (Morgan, 1889)
*Coccus hesperidum* Linnaeus, 1758
*Diaspis bouisduvali* Signoret, 1869
*Diaspis bromeliae* (Kerner, 1778)
*Diaspis echinocacti* (Bouché, 1833)
*Dynaspidiotus britannicus* (Newstead, 1896)
*Etiennea villiersi* Matile-Ferrero, 1984
*Eucalymnatus tessellatus* (Signoret, 1873)
*Gascardia hodgsoni* Matile-Ferrero & Le Ruyet, 1985
*Geococcus coffeae* Green, 1933
*Hemiberlesia rapax* (Comstock, 1881)
*Icerya purchasi* (Maskell, 1878)
*Mycetaspis personata* (Comstock, 1883)
*Nipaecoccus nipae* (Maskell, 1892)
*Prelongorthezia insignis* Browne, 1887
*Ovaticoccus agavium* (Douglas, 1888)
*Parlatoria crotonis* Douglas, 1887
*Pinnaspis aspidistrae* (Signoret, 1869)
*Pinnaspis strachani* (Cooley, 1899)
*Planococcus citri* (Risso, 1813)
*Pseudococcus longispinus* (Targioni Tozzetti, 1868)
*Pseudococcus microadonidum* Beardsley, 1966
*Pseudococcus viburni* (Signoret, 1875)
*Pulvinariella mesembryanthemi* (Vallot, 1830)
*Rhizoecus cacticans* (Hambleton, 1946)
*Rhizoecus falcifer* Künckel d’Herculais, 1878
*Saissetia coffeae* (Walker, 1852)
*Saissetia oleae* (Olivier, 1791)
*Spilococcus mamillariae* (Bouché, 1844)
*Unaspis yanonensis* (Kuwana, 1923)
*Vryburgia brevicruris* (McKenzie, 1960)

A detailed study of the scale insects introduced into Hungary on tropical and subtropical fruits was published by Kozár and Kienitz (1979), whose list already contained 13 species shown in Table 5, only one species in this category is new record for the Hungarian fauna (*Pseudococcus elisae* Borchsenius, 1957). The number of species in this category is surprisingly low, compared to the number of pests living on various fruits exported from the different regions of production. The low species number reflects the efforts made by exporting countries to prevent the spread of invasive pests. It should be noted that most of these species were unable to establish in Hungary even indoors in greenhouses or on ornamental plants in buildings, despite repeated introductions over more than one hundred years. On the other hand, some of them have become regular pests in Hungary, which has lead to some overlap with the category in Table 4. Among these species, *Aspidiotus nerii*, *Planococcus citri* and *Pseudococcus viburni* occur in greenhouses and buildings, while *Pseudaulacaspis pentagona* and *Carulaspis caruelii* are found outdoors.

**Table 5.** Scale insect species found in Hungary on imported (mainly subtropical and tropical) fruits for consumption.<br/>

*Aonidiella aurantii* (Maskell, 1879)
*Aspidiotus nerii* Bouché, 1833
*Carulaspis caruelii* (Signoret, 1869)
*Chrysomphalus aonidum* (Linnaeus, 1758)
*Chrysomphalus dictyospermi* (Morgan, 1889)
*Dysmicoccus brevipes* (Newstead, 1891)
*Lepidosaphes beckii* (Newman, 1869)
*Lepidosaphes gloverii* (Packard, 1869)
*Parlatoria pergandii* Comstock, 1881
*Parlatoria ziziphi* (Lucas, 1853)
*Planococcus citri* (Risso, 1813)
*Pseudaulacaspis pentagona* (Targioni Tozzetti, 1886)
*Pseudococcus elisae* Borchsenius, 1957
*Pseudococcus viburni* (Signoret, 1875)
